# Comparative transcriptomic analysis primarily explores the molecular mechanism of compound eye formation in *Neocaridina denticulata sinensis*

**DOI:** 10.1186/s12864-024-10453-5

**Published:** 2024-06-06

**Authors:** Congcong Yan, Zixuan Wu, Yujie Liu, Yuying Sun, Jiquan Zhang

**Affiliations:** 1 School of Life Sciences/Hebei Basic Science Center for Biotic Interaction, Hebei University, Baoding, 071002 China; 2 Institute of Life Science and Green Development, Hebei University, Baoding, 071002 China

**Keywords:** *Neocaridina denticulata sinensis*, Compound eye, Embryonic development, Comparative transcriptomic analysis, Differentially expressed genes

## Abstract

Compound eyes formation in decapod crustaceans occurs after the nauplius stage. However, the key genes and regulatory mechanisms of compound eye development during crustacean embryonic development have not yet been clarified. In this study, RNA-seq was used to investigate the gene expression profiles of *Neocaridina denticulata sinensis* from nauplius to zoea stage. Based on RNA-seq data analysis, the phototransduction and insect hormone biosynthesis pathways were enriched, and molting-related neuropeptides were highly expressed. There was strong cell proliferation in the embryo prior to compound eye development. The formation of the visual system and the hormonal regulation of hatching were the dominant biological events during compound eye development. The functional analysis of DEGs across all four developmental stages showed that cuticle formation, muscle growth and the establishment of immune system occurred from nauplius to zoea stage. Key genes related to eye development were discovered, including those involved in the determination and differentiation of the eye field, eye-color formation, and visual signal transduction. In conclusion, the results increase the understanding of the molecular mechanism of eye formation in crustacean embryonic stage.

**Supplementary Information:**

The online version contains supplementary material available at 10.1186/s12864-024-10453-5.

## Introduction

*Neocaridina denticulata sinensis* is a small freshwater shrimp with the advantages of short life cycle, easy to breed, and strong adaptability [1, 2]. Benthic lifestyle of *N. denticulata sinensis* also makes it a tool for algae removal tool [3]. According to related literature of morphological characteristics during embryonic development, the embryonic development of *N. denticulata sinensis* could be divided into 8 stages, namely one cell stage, cleavage stage, blastocyst stage, gastrula stage, nauplius stage, compound eye pigment formation I, compound eye pigment formation II and zoea [4–6]. Many important biological processes occur after the nauplius stage, such as organogenesis. Compared with other shrimp such as *Litopenaeus vannamei* and *Exopalaemon carinicauda* [7, 8], there are few reports related to embryonic development. Besides, some studies are focused on the effects of heavy metal stimulation and pathogenic bacteria stimulation on innate immune of adult shrimp [9]. Nevertheless, the molecular mechanisms and functional genes about early embryonic development of the shrimp are rarely reported. Due to its excellent ecological value and physiological features, it is urgent to study the molecular mechanisms of many important physiological changes during the embryonic development.

**Fig. 1:**
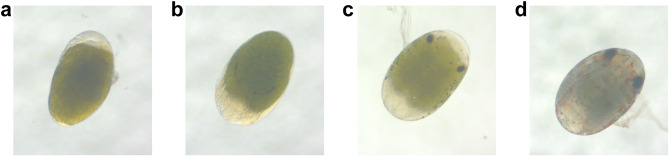
Late stage of embryonic development of *N. denticulata sinensis.* (**a**)-(**d**): Nauplius stage, Compound eye pigment formation I, Compound eye pigment formation II, Zoea stage

The appearance of crescent-shaped black thin lines on both sides of the embryo is the typical symbol of the development of compound eye pigment formation I (Fig. 1). As the embryo develops, symmetrical membranous structures appear on the medial side of the compound eyes, which gradually thicken and further develop into eyestalks. Compared with compound eye pigment formation I, the embryo with compound eye pigment formation II has the following characteristics: oval eyes, reduced yolk granules, increased muscle mass and cuticular pigment granules and more stable heart rate [4, 5]. During the zoea stage, the embryonic development is basically completed, and the morphology of the juvenile within egg membrane is like that of the adult. The embryo is translucent, with only a few yolk granules distributed on the dorsum of cephalothorax. The compound eyes are enlarged and surrounded by black radial stripes [4, 5] (Fig. 1). During embryogenesis of crustaceans, embryonic molting occurs within the embryonic envelope after the eyestalk appears. The biological process has been thought to be as hormonally regulated as adult molting [10, 11].

As the important sensory organ, compound eyes consist of numerous ommatidia, the number of which varies markedly in different arthropods [12, 13]. The functional diversity of eye is reflected in the anatomical structure of the eye and the connections with the optic lobes [14, 15]. Every ommatidium has an independent optical system, including a refractive system and a photosensitive system [14]. The refractive system consists of the cornea, corneal cells and crystalline cone. Photoreceptors are major component of the photosensitive system. During the embryonic development, the number of ommatidia increases and the surface of the ommatidium enlarges. The size and arrangement of photoreceptor cells also change [14]. During the development of compound eyes, the photoperceptivity is acquired earlier than imaging ability. The ultrastructure of compound eyes before and after hatching in *Macrobrachium nipponense* has showed that ommatidia of zoea I have photosensitivity but no imaging ability [16]. The quantity of visual opsins determines the spectral sensitivity of photoreceptors. For example, each compound eye of *L. vannamei* is composed of about 55,000–80,000 ommatidia and the highest number of visual opsin genes (42 opsin genes) is identified among animals [17]. Only two *opsins* have been identified in the *Parhyale hawaiensis* [18]. Except for the anatomy of the eyes, little is currently known about related neural circuits. Visual conduction pathways are gradually being uncovered, such as phototransduction and retinal screening pigment movements regulated by neuropeptides [19, 20]. The prevailing hypothesis about the visual system formation is the ontogenetic mechanisms that are evolutionarily homologous in Branchiopoda, Malacostraca, and Insecta [21]. Retinal determination network is an ancient set of instructions that has evolved since the emergence of primitive eyes. Highly conserved selection genes first identified in *Drosophila melanogaster* form the retinal determination gene network (RDGN) to direct eye development, and the Pax6 homolog *eyeless* is the master regulator which has also been validated in other crustaceans [13, 22]. For eye pigmentation, both ommochromes and melanins are visual pigments that are obtained sequentially from tryptophan and tyrosine through catabolic reactions [23]. During the catabolic process, eye pigment transporters are crucial to the precise transport of precursors. The ATP-binding cassette transporter subfamily G (ABCG) is initially recognized in *D. melanogaster* as the eye pigment transporters, including White, Scarlet and Brown [24]. In contrast to insects, *brown* is absent on some crustacean genomes and crustacean ABCGs are less well studied [25]. Previous researches on the visual system of crustaceans are mostly focused on multiple functions through various complex regulatory mechanisms in adults, however, there is few reports about the visual system during embryonic development.

By using the high-throughput sequencing, this study was performed to explore potential functional genes participating in visual system development during compound eye formation. Furthermore, comparative transcriptomic analysis of four different embryonic developmental stages was performed to illustrate the molecular basis of the compound eye development during embryonic development of crustaceans.

## Materials and methods

### Embryos sampling

Adult shrimps were collected from the Baiyangdian Lake in Baoding City, Hebei Province, China, and reared in indoor recirculating aquaculture systems with adequate aeration. The *N. denticulata sinensis* samples of different development stages were collected from female shrimps spawning. A total of twelve samples were collected based on their development stages: nauplius (N), compound eye pigment formation I (CE1), compound eye pigment formation II (CE2) and zoea (Z) (Fig. 1). These samples (3 × replicates for N; 3 × replicates for CE1; 3 × replicates for CE2; 3 × replicates for Z) were divided into four groups. About 50 embryos were gathered at each stage identified by observing with Stereo Microscope. Samples were immediately preserved in liquid nitrogen and then stored in -80 °C for assays.

### RNA extraction, cDNA library construction and sequencing

Total RNA was extracted from the samples using TRIzol Reagent (Invitrogen, Carlsbad, CA, USA) following the manufacture’s instruction. Total amounts and integrity of RNA were assessed using the RNA Nano 6000 Assay Kit of the Bioanalyzer 2100 system (Agilent Technologies, CA, USA). Total RNA was used as input material for the RNA sample preparations. In short, mRNA was purified from total RNA by using poly-T oligo-attached magnetic beads. mRNAs were randomly fragmented using the NEB Fragmentation Buffer and RNA-Seq libraries were constructed with the NEBNext®Ultra™ RNA Library Prep Kit for Illumina® (NEB, USA) according to the manufacturer’s instructions. In addition, the libraries were tested to ensure their qualities. Subsequently, all libraries were sequenced by the Illumina NovaSeq 6000 system (Illumina, USA) based on the basic principle of sequencing by synthesis and paired-end reads were generated.

### Transcriptome assembly, gene function annotation and quantification of gene expression

The high-throughput sequencer output were converted into raw reads by CASAVA base recognition. Raw reads were preprocessed by removing adaptors, reads with ploy-N and low-quality reads (Qphred < 20 was more than 50%). Meanwhile, Q20, Q30 and GC content of clean reads were calculated. Clean reads with high quality would be used for subsequent analyses. Reference genome and gene annotation files of *N. denticulata sinensis* were provided by our research group [26, 27]. Index of the reference genome was built using Hisat2 (v2.0.5) with default parameters. Paired-end clean reads were aligned to the reference genome using Hisat2 (v2.0.5) with *dta* and other parameters set default. After comparison, StringTie (v1.3.3b) with merge parameter to add was performed to assemble the mapped reads and generate a non-redundant, global and integrated transcript set containing known transcripts and novel transcripts across RNA-Seq samples [28]. If the gene has multiple transcripts, the longest transcript was select as the gene sequence. Novel transcripts were detected by discovering transcriptional regions without annotation information in the genome. The resulting transcripts were termed as unigenes.

Unigene function was annotated based on the following databases: COG (Cluster of Orthologous Groups of proteins), PFAM (Protein family), GO (Gene Ontology), KO (KEGG Orthology) and KEGG (Kyoto Encyclopedia of Genes and Genomes). Eggnog-mapper was used for COG annotation of assembled genes [29]. The featureCounts (v1.5.0-p3) was used to count the reads numbers mapped to each gene. Then, FPKM of each gene was calculated based on the length of the gene and reads count mapped to this gene (FPKM > 1). Based on the FPKM values, Pearson correlation analysis was performed in R package.

### Differentially expressed genes (DEGs) analysis and functional enrichment

All read counts were normalized by the TMM (Trimmed Mean of M-values) normalization method and then differential expression analysis of two groups was performed using the edgeR R package (3.24.3). The P values were calculated by the Negative binomial distribution model and adjusted using the BH (Benjamini & Hochberg) method [30]. Genes with an adjusted P-value < 0.05 and |log2(foldchange)| >= 1 were assigned as differentially expressed. We performed a Gene Ontology (GO) and KEGG pathways enrichment analysis of DEGs using the clusterProfiler R package (3.8.1). The FPKM values of unigenes were extracted and conducted log2 conversation normalized, then loaded into TBtools software to construct heatmaps in this article [31]. For the cluster analysis of DEGs, the identification of the optimal number of clusters (k) was obtained by analyzing the FPKM values of DEGs using the Elbow method. All DEGs were divided into clusters using the Mfuzz package, and the KEGG enrichment analysis was performed on the genes in each cluster. The heatmap was drawn using the ClusterGVis package.

### Phylogenetic analysis

Opsin amino acid sequences of other species were downloaded from NCBI Nr database (https://www.ncbi.nlm.nih.gov/). The opsins of *Homo sapiens* and *Euperipatoides kanangrensis* were classified as the outgroups. The detailed information was shown in Table S1. Multi-sequence alignments were performed using MAFFT software and analyzed using the maximum likelihood (ML) strategy implemented by the IQ-TREE [32]. The substitution models were predicted under default settings and Gaps/Missing Data Treatment = “Partial deletion”. Finally, the ML tree was reconstructed by using the LG + G + F settings with 1000 bootstrap replications. The resulting phylogenetic tree was visualized and modified in iTOL v6 [33].

### Validation using quantitative real-time PCR

Quantitative real-time PCR (qPCR) analysis was used to verify our transcriptome sequencing results. Eight DEGs were chosen to perform qRT-PCR and 18 S rRNA gene was chosen as an internal reference gene. Primers for qPCR were shown in Table 1. qPCR was conducted with 2 × Universal SYBR Green Fast qPCR Mix (ABclonal Technology, China) on the BIOER Real-time PCR System (BIOER, China). The PCR program was 95 °C for 3 min, then 34 cycles of 95 °C for 10 s, 55 °C for 10 s, and 72 °C for 15 s. The relative quantitative method (2^−ΔΔCT^) was used to calculate the fold change in the expression levels of chosen genes.

**Table 1:** Primers for qRT-PCR

Gene id	Annotation	Forward primer (5′ to 3′)	Reverse primer (5′ to 3′)
evm.TU.CTG_11911.44	Arrestin	CTTGACCGTGATCTCTACCTCCAT	TGGTCATGGTGACTTCCACATGCT
evm.TU.CTG_27753.12	TRPL	TCATGACCATGATGCTCCGACTTG	AGAGACCTTCGGAAATCAGCTGAG
evm.TU.CTG_49384.5	Arrestin	TCAAGTGTGCCATCGTCCAGCACAT	CTGCAGGTTGCAACCAGGAGTGATG
evm.TU.CTG_49487.9	DAG	CTACTGTCACCATTGCACAGATCT	AGCGACACTTGAACATGGTGAGAC
evm.TU.CTG_50650.9	TRP	AGGATGCACTTGAACAGACTCAAGT	CTTCATACCAGATGGATGCTAGTAG
evm.TU.CTG_15779.12	PLC-beta-4	TGATGATGACGATGCGAGAGAGGA	AGCGAGGGCTGCGACACAGGGGTT
evm.TU.CTG_29246.4	eyeless	CTAGCGCATTCAGGAGCTAGACCT	TATCGAGCCGGTCTCGTAGTACCT
evm.TU.CTG_34518.16	CePPEF	CTCACTACGCAGTCCATACACCAA	ACTCGCACTCCCTCTCTCAAAATC

Note: TRPL, transient-receptor-potential-like protein; DAG, Diacylglycerol kinase theta; TRP, transient receptor potential-gamma protein; PLC-beta-4, Phospholipase C-beta-4; CePPEF, Serine/threonine-protein phosphatase with EF-hands pef-1

## Results

### Transcriptome sequencing and assembling based on reference genome

The twelve cDNA libraries were constructed through Illumina Nova seq 6000 platform. A total of 539,421,400 raw reads were obtained respectively. After data filtering, 522,003,548 clean reads (96.77%) and 78.30 Gb clean bases were acquired. About 5.82–7.18 Gb clean bases were produced for each library. The detailed information was shown in Table S2. Mapping to the reference genome of *N. denticulata sinensis*, 70% of clean reads on average were aligned to the reference genome and 68% of reads uniquely aligned to the reference genome (Table S3). The distribution of reads in the genomic region indicated that the proportion of reads aligned to exon was the highest, followed by the intergenic region and intron (Fig. S1). Finally, the spliced clean reads were clustered into 37,185 unigenes (27,905 known unigenes, 9,280 novel unigenes).

### Functional annotation of genes and quantification of gene expression

**Fig. 2:**
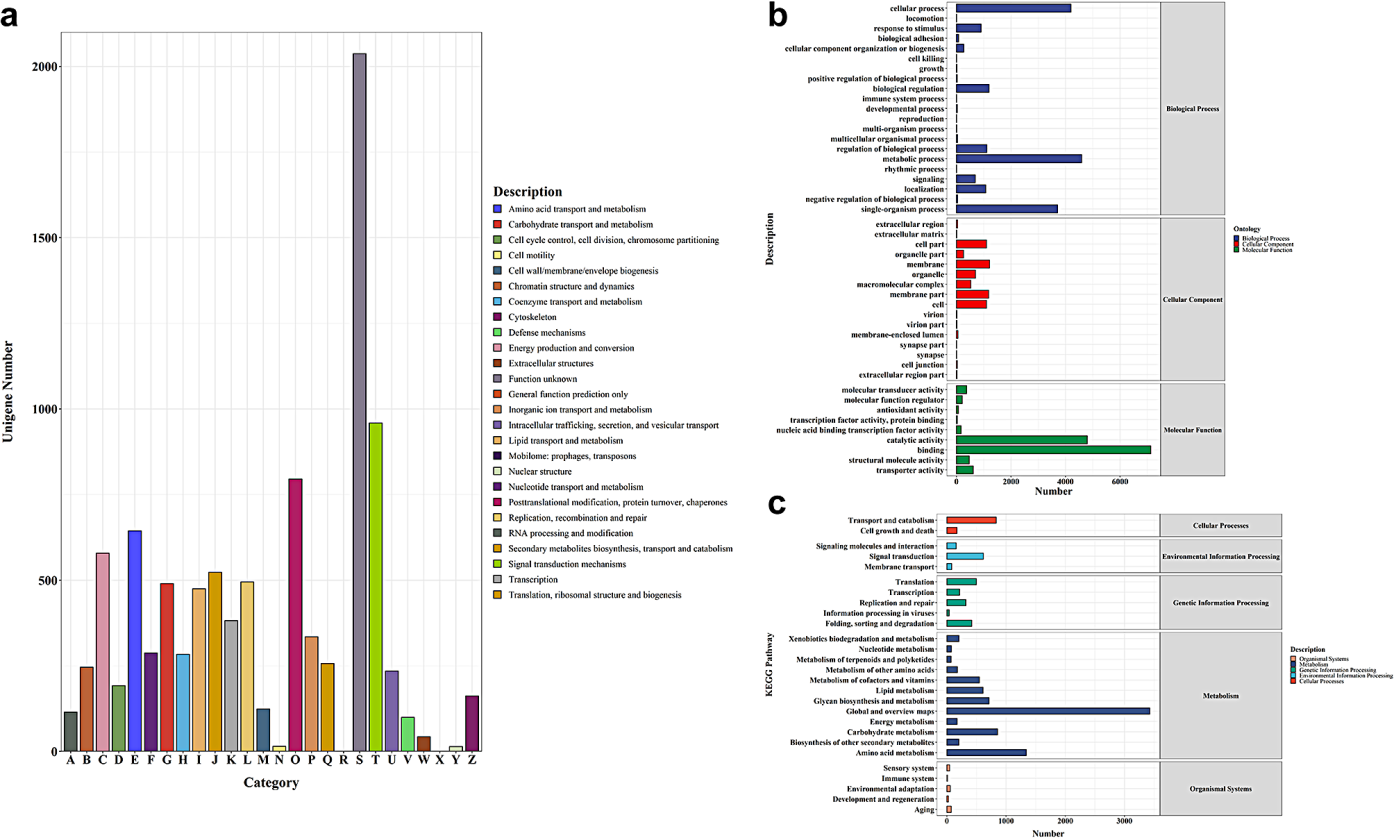
Summary of gene annotation. (**a**) The COG annotation of assembled genes. The x-axis indicates content of each category of COG and the y-axis indicates number of genes annotated in each category. (**b**) The GO annotation of assembled genes. the x-axis indicates number of genes annotated in each term and the y-axis indicates names of each term. (**c**) The KEGG classification of assembled genes

Five functional databases were performed to annotate the assembled 37,185 unigenes functionally and the results were as follows: PFAM (16,302 unigenes), GO (13,239 unigenes), COG (9,064 unigenes), KEGG (4,920 unigenes) and KO (4,750 unigenes). The annotation of known genes and new genes was shown in Table 2. A total of 9,064 unigenes (24.38%) were classified into 24 COG functional categories (Fig. 2a, Table S4). The function unknown (2,037 unigenes, 22.47%) was the predominant group, followed by signal transduction mechanisms (959 unigenes, 10.58%), posttranslational modification, protein turnover and chaperones (795 unigenes, 8.77%) (Table S4). No unigenes were functionally annotated to the remaining groups: X (Mobilome: prophages, transposons) and R (General function prediction only).

According to GO annotation analysis, 13,239 unigenes (35.60%) were classified into three functional categories: biological process, cellular component and molecular function (Fig. 2b). The result showed that membrane (GO:0016020), metabolic process (GO:0008152) and binding (GO:0005488) were the most prominent enriched terms in cellular component, biological process, and molecular function respectively (Table S5). Based on KEGG database, 4,920 (13.32%) unigenes were assigned to 27 pathways covering five major KEGG categories (Fig. 2c). Among them, transport and catabolism (834 unigenes, 16.95%), signal transduction (617 unigenes, 12.54%), translation (499 unigenes, 10.14%), global and overview maps (3428 unigenes, 69.67%) and organismal systems (75 unigenes, 1.52%) were separately the main enriched pathways in cellular processes, environmental information processing, genetic information processing, metabolism and organismal systems (Table S6).

**Fig. 3:**
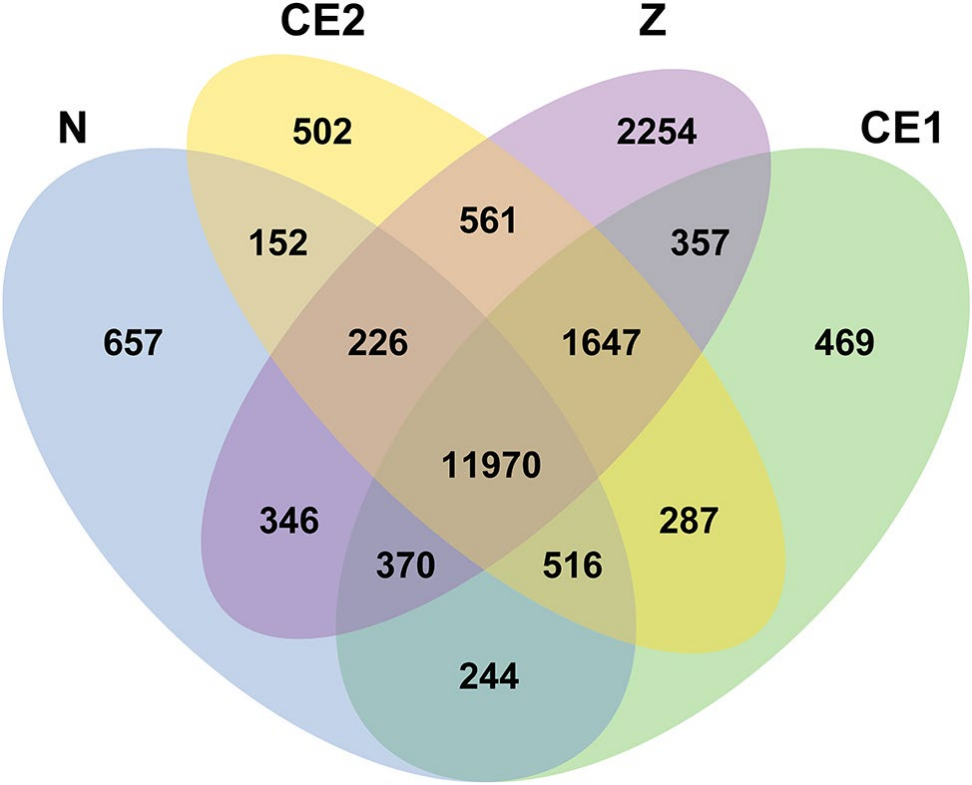
Venn diagram of expressed genes across four groups

To further explore the genes and pathways related to compound eye development, FPKM were used to show the gene expression. 11,970 co-expression genes were detected at > 1 FPKM in all four groups, while 657, 469, 502 and 2254 stage-specific genes were identified in N, CE1, CE2 and Z stages respectively (Fig. 3). The correlation of samples was evaluated by Pearson correlation coefficient (Fig. S2). The result revealed that CE1 and CE2 stage had similar expression patterns compared to N and Z stage.

**Table 2:** Summary of function annotation of *N. denticulata sinensis* transcriptome

	Number of Known Genes	Number of Novel Genes	Total Genes
Annotated in PFAM	15,638	664	16,302
Annotated in GO	12,435	804	13,239
Annotated in KEGG	4,744	176	4,920
Annotated in COG	8,855	209	9,064
Annotated in KO	4,580	170	4,750

### Identification and clustering analysis of differentially expressed genes (DEGs)

**Fig. 4:**
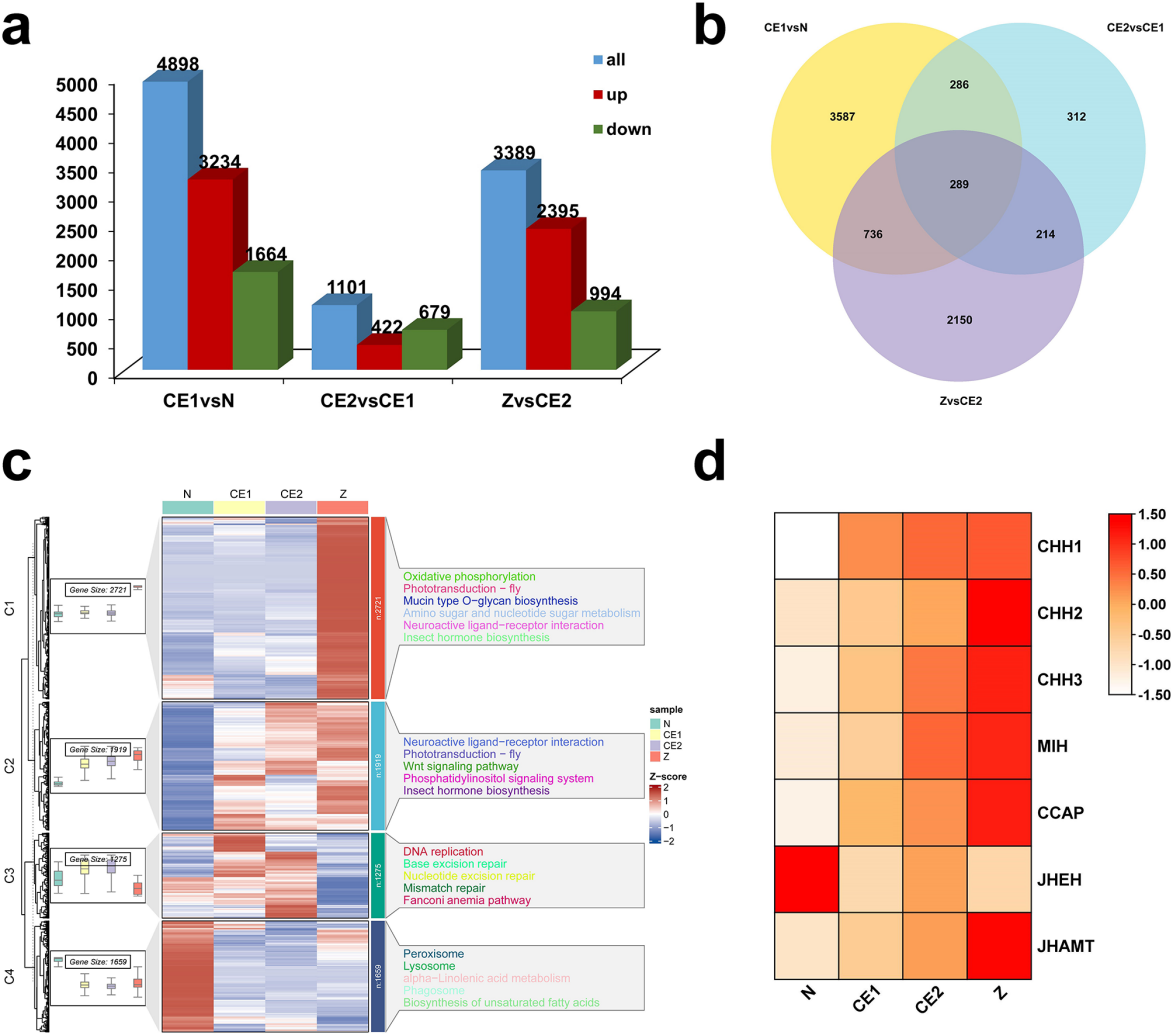
Summary of differential gene analysis. The average FPKM values were used to plot heatmaps. (**a**) Statistics of differentially expressed genes (DEGs) between adjacent groups. Blue columns represent the total number of differentially expressed genes, red columns represent up-regulated genes and green columns represent down-regulated genes. (**b**) Overlap of DEGs between comparison groups. (**c**) The clustering analysis of DEGs. The figure consists of a boxplot, heatmap and KEGG annotation information. The boxplot and heatmap shows the gene expression in each cluster, while the pathways are shown on the right side of the heatmap. For the heatmap, red indicates high relative expression level and blue indicates low relative expression level. (**d**) Heatmap of genes related to molting. CHH, crustacean hyperglycemic hormones; MIH, molt-inhibiting hormone; CCAP, crustacean cardioactive peptide; JHEH, juvenile hormone epoxide hydrolase; JHAMT, juvenile hormone acid O-methyltransferase

To explore the dynamic expression changes of genes, We compared FPKM values of genes between adjacent groups. A total of 7,574 DEGs were differentially expressed. Among them, 4,898 genes showed differential expression in CE1 vs. N group (3,234 up-regulated, 1,664 down-regulated), 1,101 DEGs in CE2 vs. CE1 group (422 up-regulated, 679 down-regulated), and 3,389 DEGs in Z vs. CE2 group (2,395 up-regulated, 994 down-regulated) (Fig. 4a). Furthermore, 289 genes were differentially expressed in all these comparison groups (Fig. 4b). In general, the comparison of N and CE1 stage had the largest number of DEGs, indicating that the transition from N to CE1 stage might be a critical stage for normal embryonic development.

According to the expression trend of DEGs, genes were divided into four clusters. The results of the cluster analysis were presented in Fig. 4c. A total of 2,721 DEGs were clustered into cluster1 (C1) and had the highest expression on Z. Except for N, the 1,919 DEGs in C2 were significantly expressed in the other three developmental stages, with the most highly expressed genes in Z stage followed by CE2 and CE1. The C3 and C4 comprised 1,275 and 1,659 genes respectively. The DEGs in C3 were highly expressed in CE1 and CE2, whereas the DEGs in C4 were only highly expressed in N stage. KEGG enrichment analysis revealed that the phototransduction - fly and insect hormone biosynthesis pathway were pathways to which both C1 and C2 were enriched. The DEGs in C3 were mainly enriched in pathways that maintained normal DNA replication, whereas the DEGs in C4 were mainly enriched to lipid metabolism-related pathways.

The insect hormone biosynthesis pathway was related to the synthesis of juvenile hormones and ecdysteroids, and the hatching is like eclosion in insects [10, 34]. Therefore, we hypothesized that the insect hormone biosynthesis pathway might be related to the hormonal regulation of hatching. Since crustacean molting was regulated by multiple neuropeptides and sesquiterpenoid pathway [35, 36], we further explored key ecdysis-related members and their expression patterns during embryonic development, namely crustacean hyperglycaemic hormone (CHH), molt-inhibiting hormone (MIH), crustacean cardioactive peptide (CCAP), juvenile hormone epoxide hydrolase (JHEH) and juvenile hormone acid methyltransferase (JHAMT). The expression profile showed a significant increase in the mRNA expression levels of key neuropeptides involved in ecdysone synthesis (MIH, CHH, CCAP) and rate-limiting enzyme (JHAMT) on the JH pathway (Fig. 4d, Table S7). Therefore, it was inferred that ecdysteroid synthesis might occur along with eye development. Based on the cluster analysis, the development of the photosensitive system and the hormonal regulation of hatching might become the dynamic biological events during the development of compound eye.

### Gene ontology enrichment and KEGG pathway enrichment analysis for DEGs

**Fig. 5:**
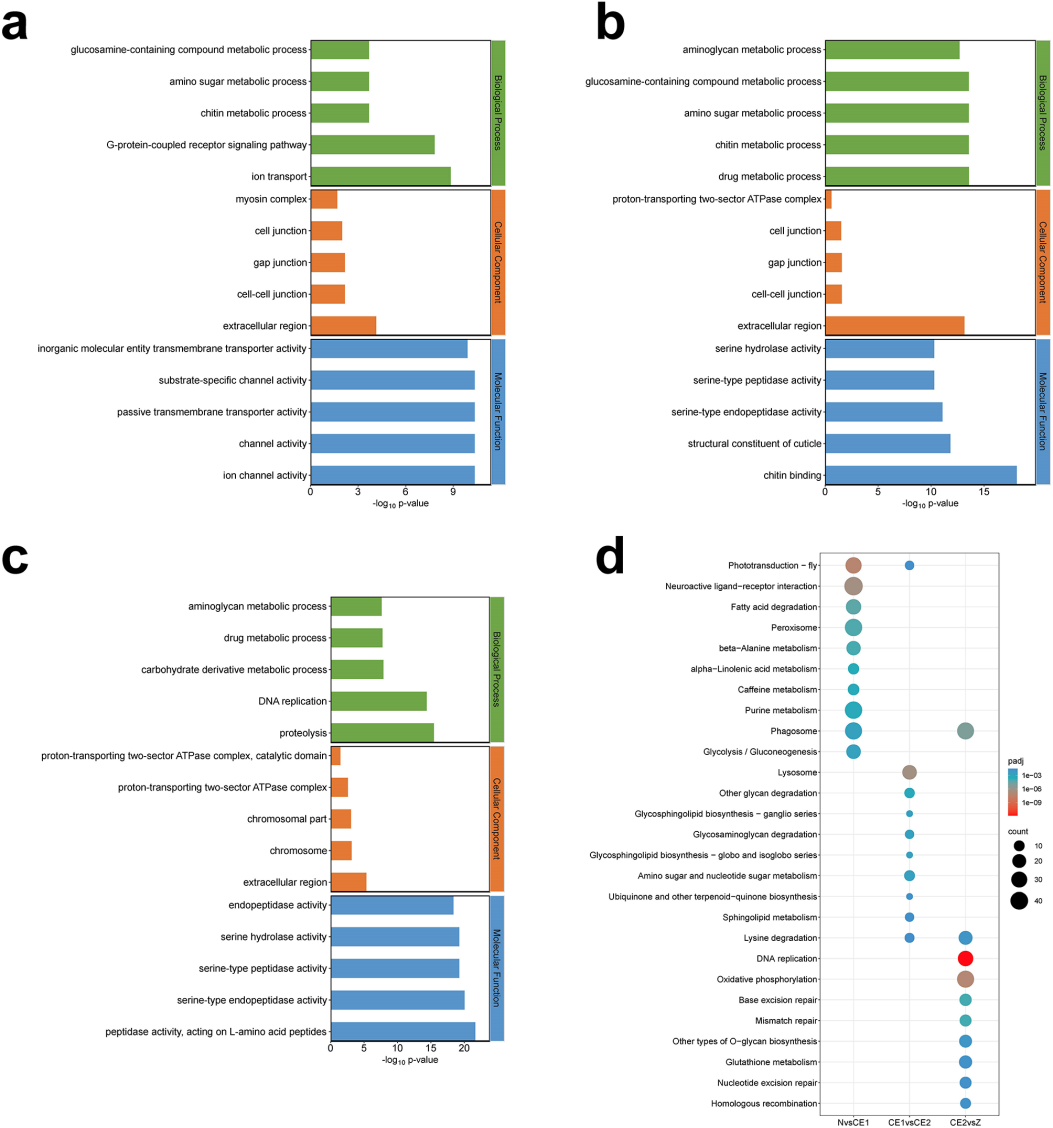
Enrichment analysis of DEGs. (**a**) GO enrichment analysis from N to CE1. X-axis indicates -log_10_ p-value, and the Y-axis indicates the enriched GO terms. Three different colors of the columns represent three basic classifications of GO term. Each category displays five GO terms. (**b**) GO enrichment analysis from CE1 to CE2 stage. (**c**) GO enrichment analysis from CE2 to Z stage. (**d**) KEGG enrichment of DEGs. X-axis indicates the comparison groups and Y-axis indicates the pathway names. The different colors of the dots represent the p-value, and the number of DEGs in each pathway is represented by the size of the dots

GO functional enrichment analysis further detected significantly enriched GO terms in DEGs (padj < 0.05). In the comparison between N and CE1 stage, 619 DEGs were significantly enriched in 44 GO terms. Ion transport (GO:0006811) and extracellular region (GO:0005576) were the most highly enriched GO terms in biological process category and cellular component category, respectively. In molecular function category, ion channel activity (GO:0005216), channel activity (GO:0015267), passive transmembrane transporter activity (GO:0022803) and substrate-specific channel activity (GO:0022838) were the most enriched four GO terms (Fig. 5a). However, 170 DEGs were significantly enriched in 22 GO terms between CE1 and CE2 stage. The top four enriched GO terms of biological processes were related to metabolic processes comprising drug metabolic process (GO:0017144), chitin metabolic process (GO:0006030), amino sugar metabolic process (GO:0006040) and glucosamine-containing compound metabolic process (GO:1,901,071). As for cellular component category and molecular function category, extracellular region (GO:0005576) and chitin binding (GO:0008061) were the most enriched subclasses, respectively (Fig. 5b). In contrast to the other two comparison groups, 619 DEGs from CE2 to Z stage, were significantly enriched in 61 GO terms, with proteolysis (GO:0006508), extracellular region (GO:0005576) and peptidase activity, acting on L-amino acid peptides (GO:0070011) were the most significant GO terms in biological process, cellular component and molecular function separately (Fig. 5c). Chitin metabolic process (GO:0006030) was common among the three comparable groups, suggesting that the cuticle of embryos might be being constructed.

**Fig. 6:**
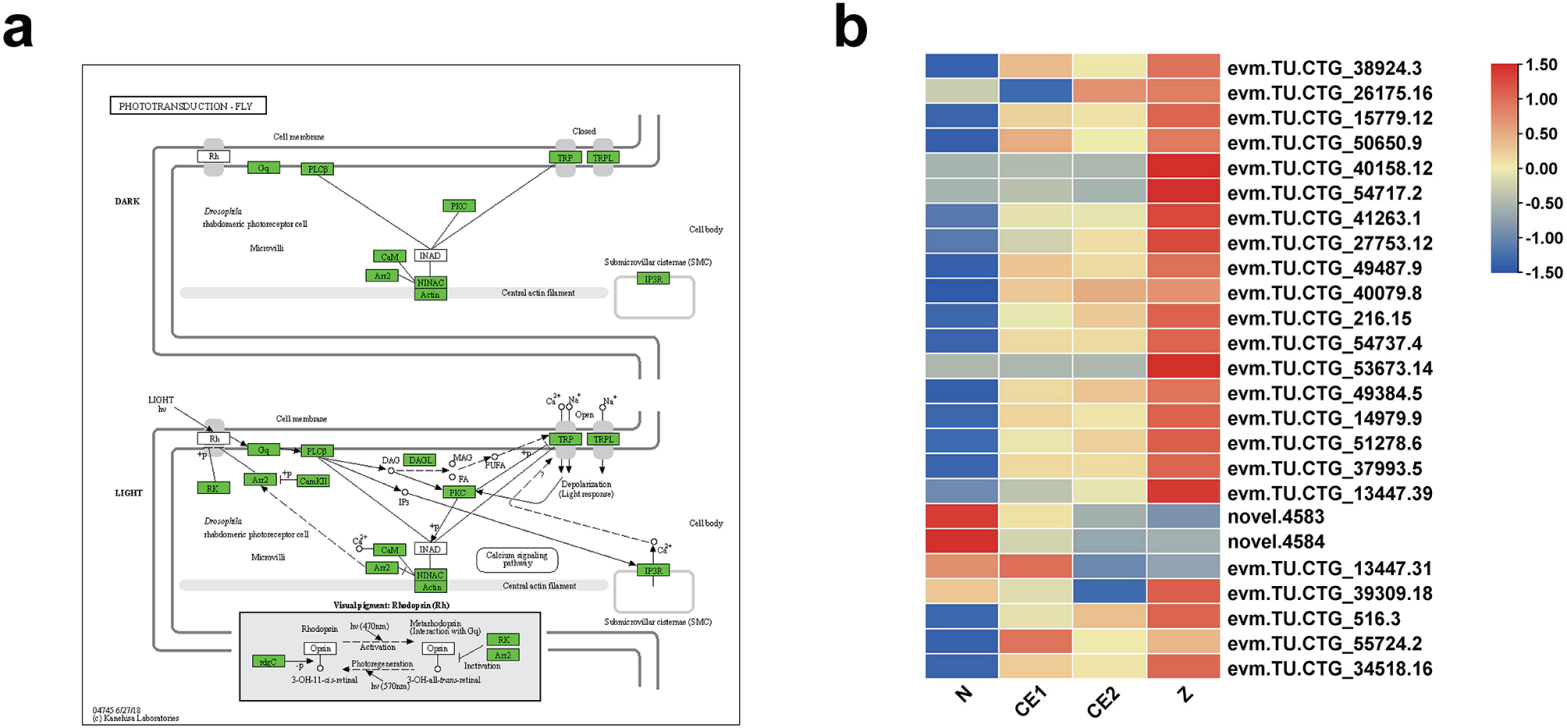
The summary of the phototransduction - fly signaling pathway. The average FPKM values were used to plot heatmaps. (**a**)The KEEG pathway of the phototransduction - fly signaling pathway. The green background indicates the unigenes annotated on the pathway. (**b**) The expression patterns of DEGs enriched in phototransduction - fly signaling pathway

Performed by KEGG, the DEGs in N-CE1 stage were mainly enriched in pathways related to amino acid metabolism and carbon metabolism like beta-Alanine metabolism (ko00410) and fructose and mannose metabolism (ko00051) (Fig. 5d, Table S8). In addition to amino acid metabolism, some pathways were significantly enriched in CE1 vs. CE2 group, including glycerolipid metabolism (ko00561), glycosaminoglycan degradation (ko00531) and sphingolipid metabolism (ko00600). For CE2 vs. Z group, some KEGG pathways were associated with genetic information processing and energy metabolism, such as DNA replication (ko03030), homologous recombination (ko03440) and oxidative phosphorylation (ko00190). Besides, visual system-related pathway (phototransduction - fly) were significantly enriched in different comparison groups. Twenty-five DEGs were enriched for the phototransduction - fly pathway, and their expression profiles indicated that proliferating cells in the eye field might be gradually differentiated into photoreceptors at CE1 stage (Fig. 6).

### Identification of shared and stage-specific DEGs at four different development stages

**Fig. 7:**
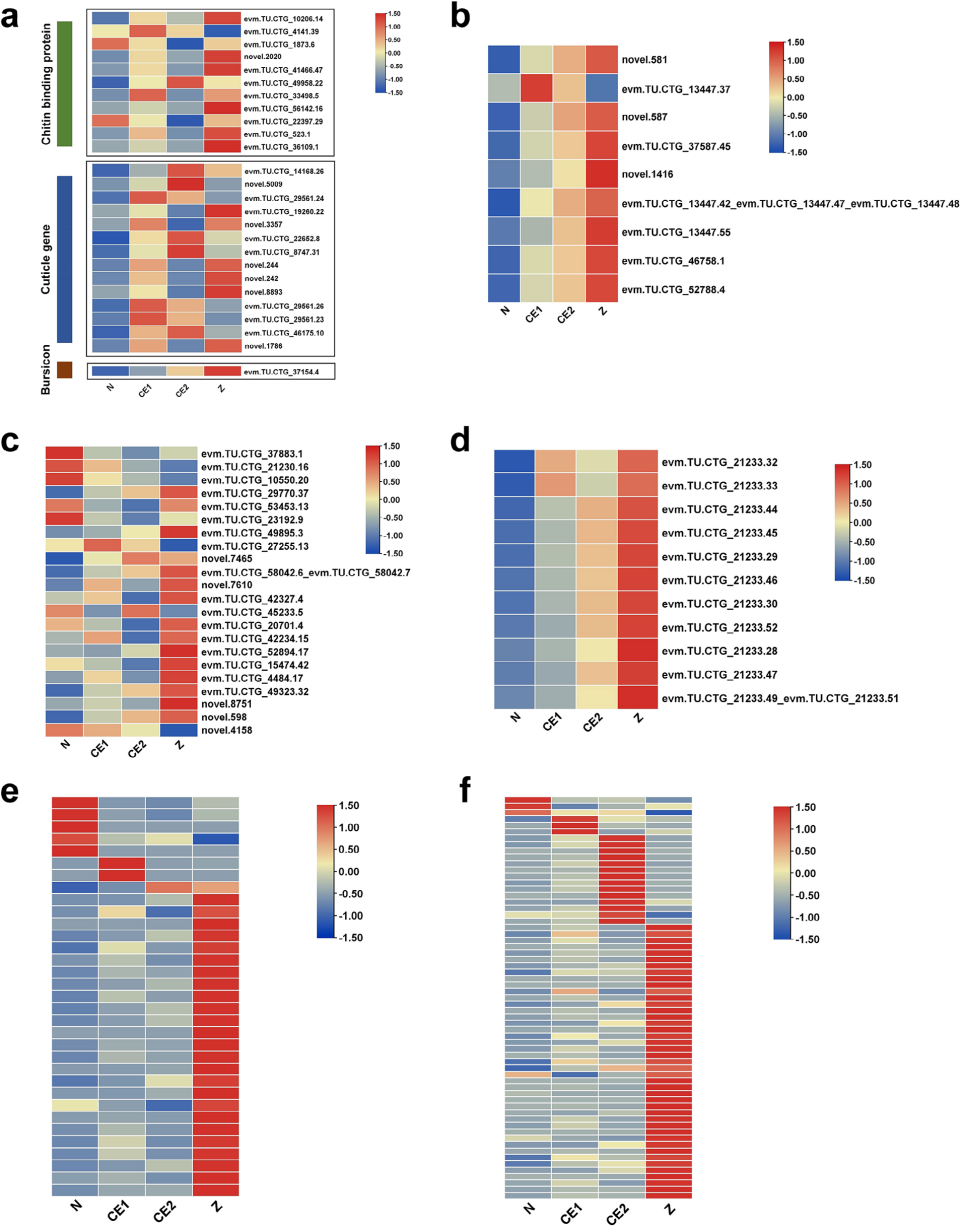
Clustering of DEGs identified in all three comparative groups. The average FPKM values were used to plot heatmaps. (**a**) Heat map of DEGs related to cuticle formation. Purple label indicates the genes related to chitin metabolism pathway, pink indicates putative cuticle gene, and green indicates the bursicon gene (**b**) Heatmap of DEGs related to muscle growth. (**c**) Heatmap of DEGs related to immune system. (**d**) Heatmap of crustacyanin genes. (**e-f**) Heatmap of stage-specific genes associated with chitin binding and structural constituent of cuticle

Among the DEGs identified, 289 genes appeared in all four different stages of embryonic development, suggesting continuous change throughout development. Functional annotation indicated that these genes were mainly involved in cuticle formation, muscle growth, and the establishment of immune system (Fig. 7). The detailed annotation information was shown in Table S9. Among the DEGs related to cuticle formation, there were eleven genes enriched in chitin metabolism pathway, fifteen genes annotated as cuticle proteins and one Bursicon gene. When the embryo developed to CE1 stage, the expression of related DEGs significantly increased and reached the maximum during Z stage (Fig. 7a). Moreover, there were nine DEGs related to muscle growth, including troponin C (2) and various types of actin (7). The results showed that, except for one transcript (evm.TU.CTG_13447.37), the expression of other genes gradually increased (Fig. 7b). The changes in the expression of these DEGs were consistent with the phenotype of appendage formation and muscle enlargement during four developmental stages. Besides, a total of 22 immune related genes were also screened, involving immune effector factors, immune recognition receptors, and signaling pathways (Fig. 7c). It indicated that the embryonic immune system was constantly improving. Three DEGs involved in phototransduction and eleven DEGs annotated as crustacyanin were found, with their expression levels continuously increasing (Fig. 7d). It was suggested that the crustacyanin genes should act in the formation of various pigment cells at later stages of embryonic development [37]. For stage-specific genes, enrichment analysis revealed that 33 and 63 stage-specific genes were associated with chitin binding (GO:0008061) and structural constituent of cuticle (GO:0042302), respectively (Fig. 7e-f, Table S10). And the number of these stage-specific genes gradually increased from N to Z stage.

### Discovery of key genes related to eye development

**Fig. 8:**
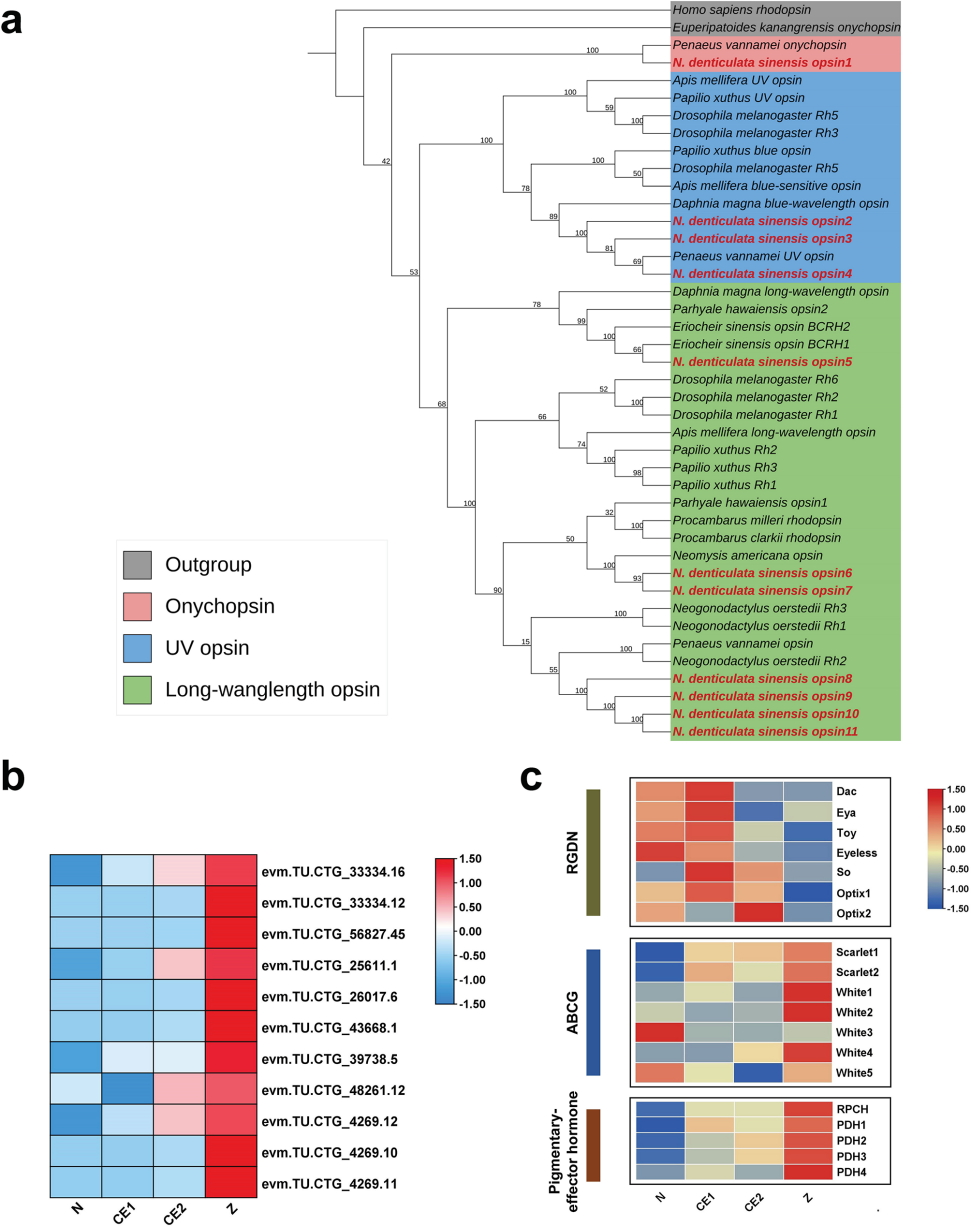
Analysis of key genes related to eye development. (**a**) Phylogenetic tree of visual opsins. The opsins of *N. denticulata sinensis* is marked in the red typeface. All accession numbers of opsin sequences used in the phylogenetic tree are shown in Table S1. (**b**) Heatmap of putative visual opsins of *N. denticulata sinensis.* The average FPKM values were used to plot the heatmap. (**c**) Putative genes related to eye determination and eye color of *N. denticulata sinensis*

Compound eye formation was a distinct and important developmental phenotype during embryonic development from N to Z stage. Therefore, it was necessary to uncover key members at the molecular level (Fig. 8, Table S11). For the determination and differentiation of the eye field, the RD network had five key members, namely *eyeless* (*ey*), *twin of eyeless* (*toy*), *sine oculis* (*so*), *eyes absent* (*eya*) and *dachshund* (*dac*). *Optix* also participated in the formation of eye morphology. Based on the sequence information of *D. melanogaster*, a total of seven candidate homologs were identified, including two *optix* homologs. In eye color, we identified two *Scarlet* and five *White* based on the transcriptome, but there was no *brown*. In terms of visual signal transduction, opsins converted absorbed photons into electrical signal through the phototransduction mechanism. In this study, 14 putative opsins were identified, including 11 visual opsins and 3 non-visual opsins (Table S11). The phylogenetic analysis showed that 11 visual opsins were classified into three categories: long-wavelength-sensitive opsins (7), ultraviolet-sensitive opsins (3), and onychopsin (1) (Fig. 8a). When the embryo developed to Z stage, the expression level of visual opsins significantly increased (Fig. 8b). To respond to environmental stimuli, pigmentary-effector hormones could regulate retinal pigment and phototransduction processes. Four putative orthologues were found in the transcriptome, namely one RPCH (red pigment-concentrating hormone) and three PDHs (pigment-dispersing hormone) (Fig. 8c). In short, the identification of aforementioned genes would help to further analyze the formation and neurogenesis of the visual system in *N. denticulata sinensis.*

### The qRT-PCR validation of DEGs

To validate transcriptome sequencing data, partial DEGs in the phototransduction pathway and RDGN were selected for quantitative real-time PCR analysis. The result of qRT-PCR showed that expression changes of the eight selected DEGs were consistent with that of the transcriptome data, which confirmed the reliability of the transcriptome data (Fig. S3).

## Discussion

*N. denticulata sinensis* has high ecological and economic value due to the biological characteristics of short life cycle and easy to breed. It has been successfully breed in laboratory [3]. However, little is yet known about molecular mechanisms of important physiological changes in early development. Therefore, it is extremely essential for the research about embryonic development. In this study, the transcriptome from N to Z stage was assembled. A lot of 7,574 DEGs were identified and classified by GO and KEGG enrichment analysis to further investigate dynamic molecular mechanisms of late embryonic development. The clustering analysis of DEGs indicated that there was strong cell proliferation and lipid consumption in the embryo prior to compound eye development, and that the formation of the visual system and the hormonal regulation of hatching became the major biological events from CE1 to Z stage.

Another important process identified by this study is cuticular formation. Cuticle consists of chitin and cuticle proteins [38]. During the crustacean embryogenesis, the loose cuticle forms after the nauplius stage and rapidly sheds after hatching [39]. In this study, chitin metabolic process (GO:0006030) and chitin binding (GO:0008061) were important terms significantly enriched in biological processes and molecular functions, respectively. The analysis about DEGs across all four different development stages showed that the expression of multiple cuticle and one Bursicon gene increased significantly with embryonic development. The number of stage-specific genes in cuticle formation also increased from N to Z stage. These results suggested that the cuticle began to form after the nauplius stage. The compound eye development is accompanied by the eyestalk organogenesis. During embryogenesis of crustaceans, embryonic molting occurs within embryonic envelopes and the hatching is similar to eclosion in insects [10, 40]. In crustaceans, molting is regulated by multiple neuropeptides, such as MIH, CHH, CCAP and Bursicon [35, 41]. MIH and CHH secreted by the eyestalk ganglia (XO-SG complex) negatively regulate ecdysone release from the Y-organ through G protein-coupled receptor-mediated signaling pathways [35]. Ecdysone and MIH in hemolymph antagonize each other to regulate the molting process [38]. Besides, CHH affects the water absorption for eggshell rupture and CCAP participates in the beginning of the molting process [10, 41]. JHEH is an important degrading enzyme regulating the titer of juvenile hormone (JH), and JHAMT has been suggested to be a rate-limiting factor in JH-signaling pathway [42]. Studies on *Daphnia magna* have shown that the sesquiterpenoid pathway has an antagonistic effect on the ecdysteroid pathway [36]. In this study, the significant expression of neuropeptides suggested the establishment of an ecdysteroid synthesis mechanism during late embryonic development. Expression profiles of *JHEH* and *JHAMT* indicated that synthesis of JH was decreased from N to Z stage. Therefore, the ecdysone titer of embryos should be increased from N to Z stage and embryonic ecdysis may occur after CE1 stage. These results will increase the understanding of the molecular mechanisms by which the endocrine system regulates embryonic development in crustaceans.

The embryo of *N. denticulata sinensis* have differentiated into muscle during N stage. Actin and myosin are important components of muscle cells. Besides, *twist* and *mef2* play key regulatory roles in myogenesis [43, 44]. Many actin transcripts were identified in this study. Their expression varied significantly between the comparison groups, which was consistent with the phenotype of the embryos. Moreover, it was found that *mef2* had a smooth expression. The signaling pathways involved in muscle development were not significantly enriched, such as Notch signaling pathway, Hedgehog signaling pathway and Wnt signaling pathway [45–47]. Therefore, it indicated that muscle development might occur smoothly compared to other biological processes from N to Z stage.

The formation of compound eyes is a distinctive feature during late stage of embryonic development. During the stage of compound eye pigment formation, melanin gradually deposits to form crescent moon-shaped thin lines on both sides of the embryo. Then, oval eyes are formed till Z stage [4]. In this study, there were some KEGG pathways related with visual system, such as phototransduction-fly. For compound eyes, ommatidia are independent photographic units, all of which work together to complete an image. It is indicated that each ommatidium contains different types of photoreceptor neurons, cone cells, pigment cells, secreted lenses and retinal cells [48–50]. Invertebrates’ photoreceptor is rhabdomeric type [50–52], and the phototransduction process occurs within its. The phototransduction is mediated by a G-protein alpha-q (Gq)-coupled phospholipase C (PLC) signaling cascade among arthropods [19, 53]. The absorbed photons are converted into electrical signals through the activation of opsin (rhodopsin), which are further presented to the optic lobe. The activation and deactivation mechanisms of the phototransduction have been elaborated in detail in *Drosophila*. Specifically, Ca^2+^ acts as control switch for the activation and deactivation of phototransduction by negatively regulating photosensitive channels (TRP and TRPL) [54]. Pathway enrichment analysis showed that a total of 59 genes were enriched in the phototransduction - fly, including 25 DEGs classified into 12 different proteins (Fig. 6, Table S11). Overall, the expression of DEGs gradually increased from N to Z stage, suggesting that the visual system was becoming fully developed.

In addition, key genes involved in eye development were also identified. Retinal determination (RD) network in *D. melanogaster* is controlled by a series of conserved genes. The core members contain 5 genes, namely *eyeless* (*ey*), *twin of eyeless* (*toy*), *sine oculis* (*so*), *eyes absent* (*eya*) and *dachshund* (*dac*) [22]. These key members control eye development by constituting the retinal determination gene network, where the upstream gene *ey* is the switch for eye development. The functional conservation of *ey* has now been validated in a variety of arthropods by knockout [13, 55, 56]. Therefore, the candidate genes in this network are beneficial for determining the onset of eye development in *N. denticulata sinensis.* In terms of visual formation, opsins play a crucial role in signal conversion and are divided into visual opsins and non-visual opsins [57, 58]. Visual opsins (rhodopsin) involve in the phototransduction process by combining with chromophores [53]. Non-visual and extraocular photoreceptors exist different tissue and perform non-imaging functions [59]. For example, the pteropsin found in the brain of honey bee plays a role in biological rhythms [60]. In this study, 11 visual opsins and 3 non-visual opsins were confirmed to be present in the transcriptome. Although phylogenetic analysis showed that 11 rhodopsins were classified into three categories, it was still necessary to determine their spectral characteristics through measurement. Besides, some studies have shown that opsins also have light-independent roles [61]. Visual opsins of *Drosophila* facilitate mechanotransduction in auditory sensory cells [62]. Therefore, the identification of opsins in the transcriptome helps to further explore the role of multifunctional sensory receptors in *N. denticulate sinensis.* For eye color-related genes, the molecular mechanism of eye pigment transporters (ABCG) is clearly elucidated in *D. melanogaster* [24, 63]. The combination of White and Scarlet produces brown pigment, while the combination of White and Brown leads to the production of red pigment drosopterin. Besides, the role of *scarlt* does not appear to be conserved in crustacean eye development. Knockdown of *scarlet* in *Daphnia magna* and *Eriocheir sinensis* separately changes eye color from black to white and red, demonstrating that *scarlet* is necessary for black pigmentation in eyes [25, 64]. However, the deletion of *scarlet* in *Neocaridina heteropoda* causes eye morphology defects [64]. Given the importance of ABCG for eye-color formation, nine potential ABCGs were identified in this study, including two *scarlet* genes and seven *white* genes. Like *D. magna*, it lacked the *brown* ortholog and had multiple *white* orthologs. Furthermore, two *scarlt* homologs were found in the *N. denticulata sinensis.* In general, the *brown* homolog seems to be insect-specific. It is currently unclear whether the number of the *white* and *scarlt* will affect eye development.

During embryonic development from N to Z stage, there was significant pigment deposition on the epidermis and eyes of *N. denticulata sinensis* (Fig. 1). Research has shown that body color is formed by the dispersion and accumulation of pigments in chromatophores. Carotenoids are the main pigments in crustaceans. Since crustaceans are unable to synthesize carotenoids, carotenoids rely on the supply of maternal yolk protein for embryos [65]. Carotenoids are converted into astaxanthin through the carotenoid metabolism pathway in crustaceans [66]. Astaxanthin and crustacyanin combine to produce body color. Therefore, the present study suggests that depletion of yolk granules could provide sufficient carotenoids to the embryo, and that the markedly elevated expression of crustacyanin gene may be intended to bind to more astaxanthin to cause pigment deposition. Carotenoid metabolic pathways enriched in the transcriptome may also be involved in astaxanthin synthesis.

## Conclusions

In summary, this study focused on the transcriptomes from N to Z stage in *N. denticulata sinensis*, with the purpose of comparative analysis of physiological changes during the formation of compound eye. 37,185 unigenes were assembled and 7,574 DEGs were also identified. The clustering analysis of DEGs indicated that the formation of the visual system and the hormonal regulation of hatching became the dominant biological events when compound eye development was initiated. By GO and KEGG enrichment analysis, DEGs were mainly mapped to pathways connected with phototransduction, lipid metabolism and cell cycle regulation, suggesting that there were compound eye development and organogenesis. The functional analysis of DEGs across all four different development stages showed that these genes were mainly involved in the cuticle formation, muscle growth, and the establishment of immune system. The identification of key genes involved in eye development based on previous literature would contribute to exploring the molecular mechanisms and evolution of eye development in crustaceans.

### Electronic supplementary material

Below is the link to the electronic supplementary material.

Supplementary Material 1

Supplementary Material 2

Supplementary Material 3

Supplementary Material 4

Supplementary Material 5

Supplementary Material 6

Supplementary Material 7

Supplementary Material 8

Supplementary Material 9

Supplementary Material 10

Supplementary Material 11

Supplementary Material 12

Supplementary Material 13

## Data Availability

Data is provided within the manuscript or supplementary information files. Raw sequence data reported in this paper have been deposited (PRJCA023852) in the Genome Sequence Archive in the BIG Data Center, Chinese Academy of Sciences under accession codes CRA015092 for transcriptome sequencing data (https://ngdc.cncb.ac.cn/gsa).
