# Supplementary material for: Comparative transcriptomic analysis primarily explores the molecular mechanism of compound eye formation in Neocaridina denticulata sinensis
Source: BMC Genomics. 2024 Jun 6;25:570. doi: 10.1186/s12864-024-10453-5 (PMC11155044; doi:10.1186/s12864-024-10453-5)
Supplement: Supplementary file 13 — Supplementary Material 13 [file 12864_2024_10453_MOESM13_ESM.pptx]

## Slide 1
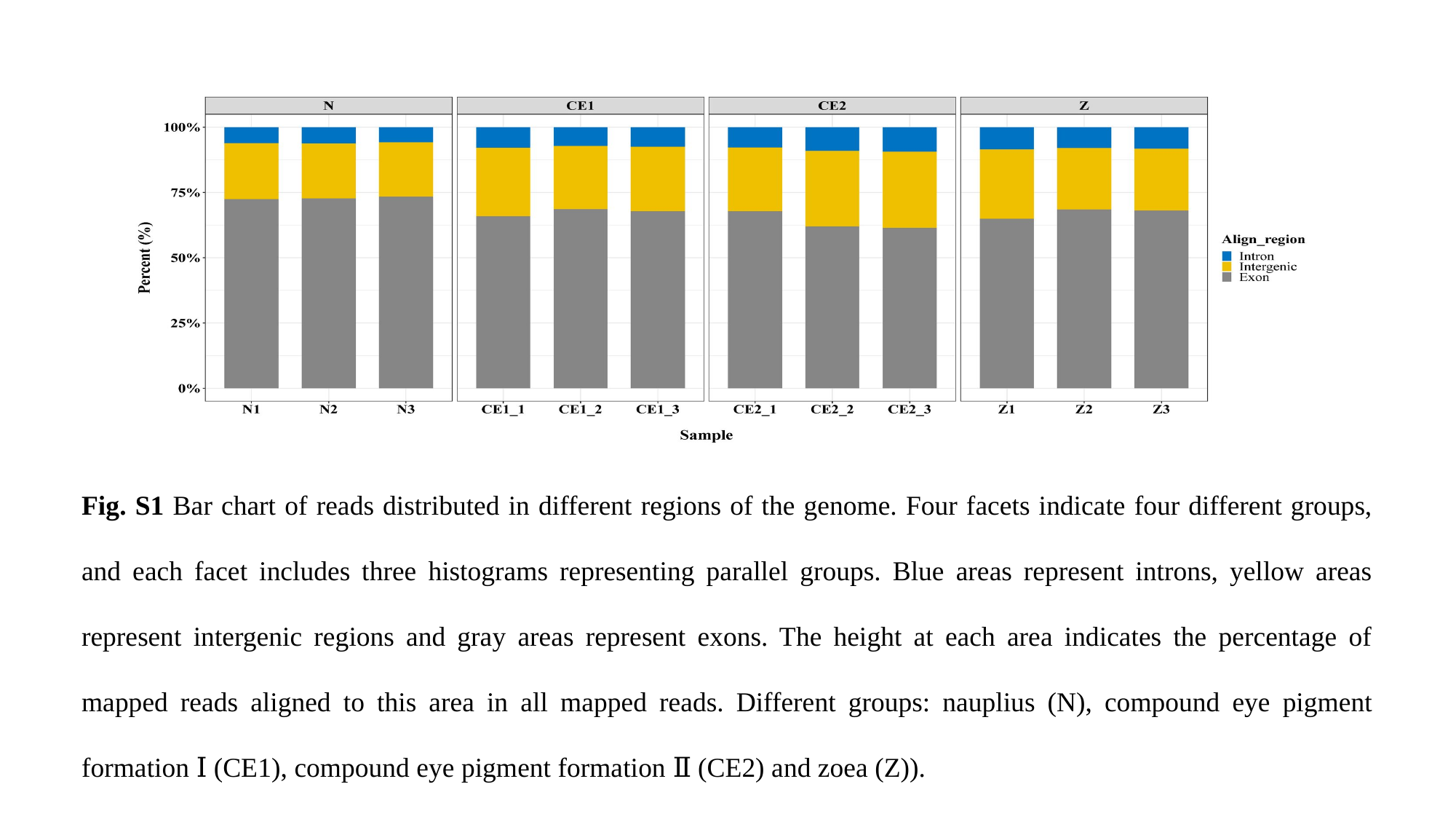

Fig. S1 Bar chart of reads distributed in different regions of the genome. Four facets indicate four different groups, and each facet includes three histograms representing parallel groups. Blue areas represent introns, yellow areas represent intergenic regions and gray areas represent exons. The height at each area indicates the percentage of mapped reads aligned to this area in all mapped reads. Different groups: nauplius (N), compound eye pigment formation Ⅰ (CE1), compound eye pigment formation Ⅱ (CE2) and zoea (Z)).

## Slide 2
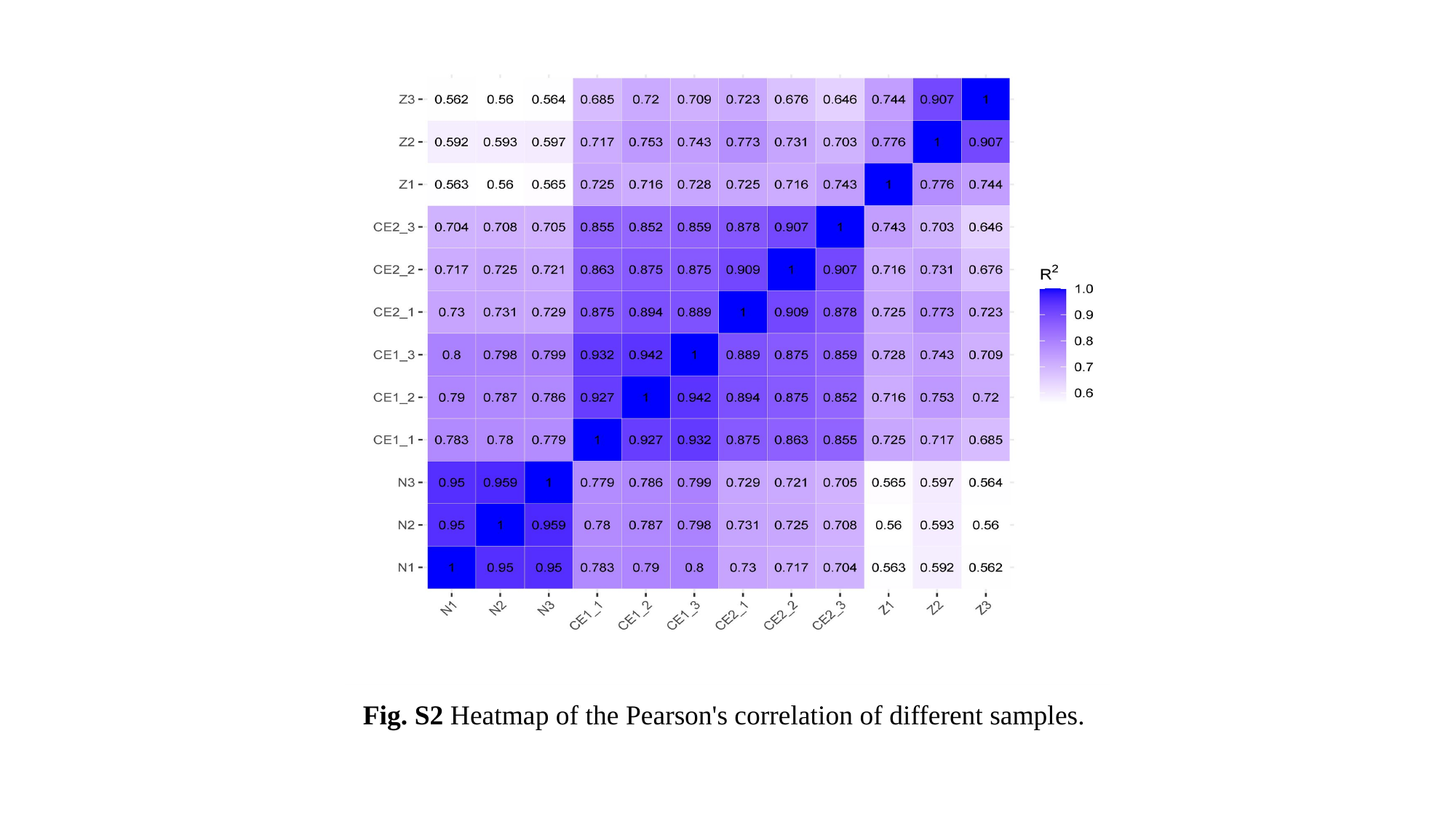

Fig. S2 Heatmap of the Pearson's correlation of different samples.

## Slide 3
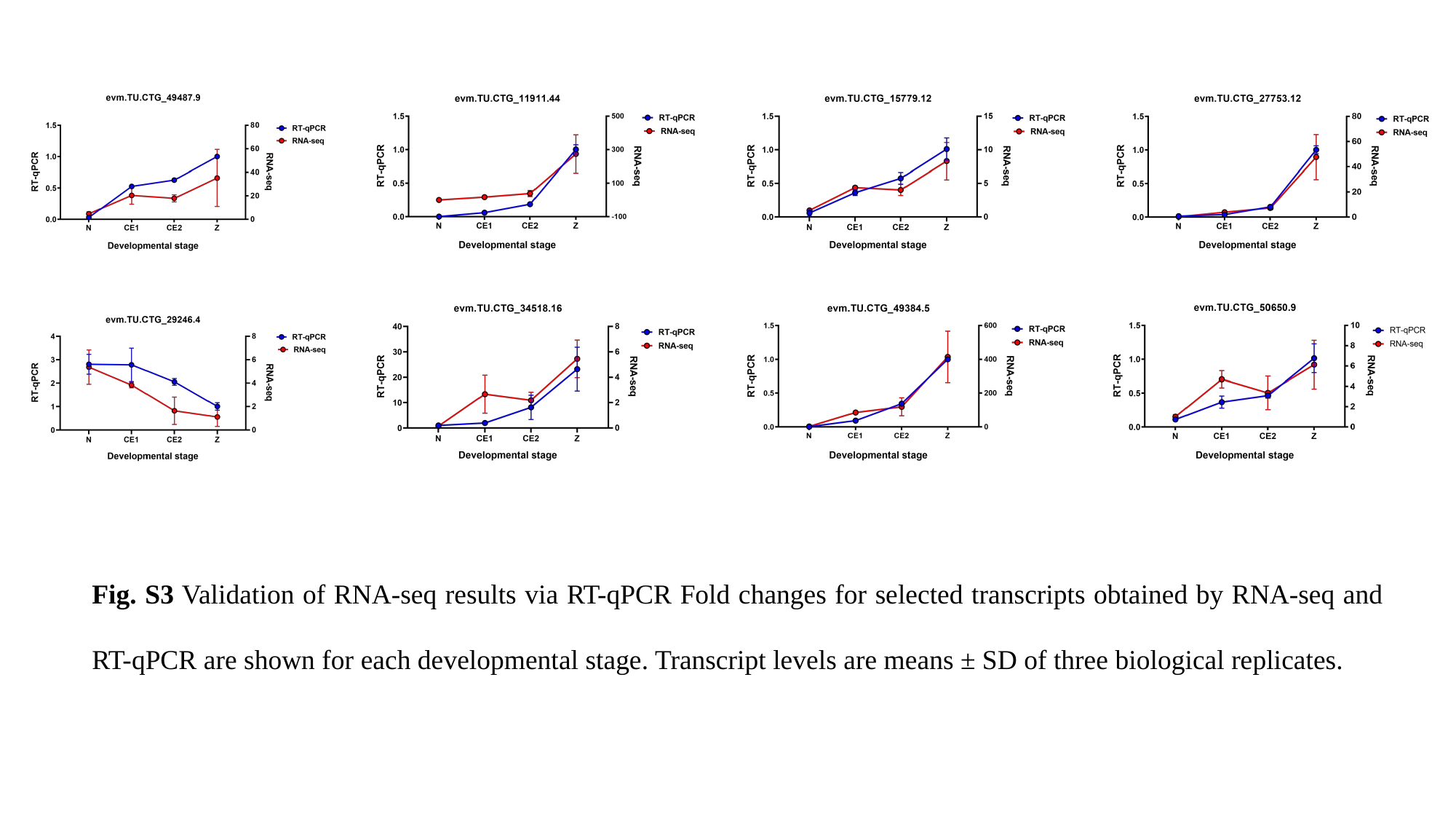

Fig. S3 Validation of RNA-seq results via RT-qPCR Fold changes for selected transcripts obtained by RNA-seq and RT-qPCR are shown for each developmental stage. Transcript levels are means ± SD of three biological replicates.
